# High Performance Acetylene Sensor with Heterostructure Based on WO_3_ Nanolamellae/Reduced Graphene Oxide (rGO) Nanosheets Operating at Low Temperature

**DOI:** 10.3390/nano8110909

**Published:** 2018-11-05

**Authors:** Zikai Jiang, Weigen Chen, Lingfeng Jin, Fang Cui, Zihao Song, Chengzhi Zhu

**Affiliations:** 1 State Key Laboratory of Power Transmission Equipment & System Security and New Technology, Chongqing University, Chongqing 400044, China; cqujlf@cqu.edu.cn (L.J.); 20161113042t@cqu.edu.cn (F.C.); 201711131056@cqu.edu.cn (Z.S.); 2 School of Electrical Engineering, Chongqing University, Chongqing 400044, China; 3 State Grid Zhejiang Electric Power Co., Ltd., Hangzhou 310000, China; 20161102053t@cqu.edu.cn

**Keywords:** WO_3_ nanolamellae, reduced graphene oxide, acetylene sensing performance, gas sensing mechanism, dissolved gases in transformer oil

## Abstract

The development of functionalized metal oxide/reduced graphene oxide (rGO) hybrid nanocomposites concerning power equipment failure diagnosis is one of the most recent topics. In this work, WO_3_ nanolamellae/reduced graphene oxide (rGO) nanocomposites with different contents of GO (0.5 wt %, 1 wt %, 2 wt %, 4 wt %) were synthesized via controlled hydrothermal method. X-ray diffraction (XRD), transmission electron microscopy (TEM), Raman spectroscopy, X-ray photoelectron spectroscopy (XPS), thermogravimetric analyses-derivative thermogravimetric analysis-differential scanning calorimetry (TG-DTG-DSC), BET, and photoluminescence (PL) spectroscopy were utilized to investigate morphological characterizations of prepared gas sensing materials and indicated that high quality WO_3_ nanolamellae were widely distributed among graphene sheets. Experimental ceramic planar gas sensors composing of interdigitated alumina substrates, Au electrodes, and RuO_2_ heating layer were coated with WO_3_ nanolamellae/reduced graphene oxide (rGO) films by spin-coating technique and then tested for gas sensing towards multi-concentrations of acetylene (C_2_H_2_) gases in a carrier gas with operating temperature ranging from 50 °C to 400 °C. Among four contents of prepared samples, sensing materials with 1 wt % GO nanocomposite exhibited the best C_2_H_2_ sensing performance with lower optimal working temperature (150 °C), higher sensor response (15.0 toward 50 ppm), faster response-recovery time (52 s and 27 s), lower detection limitation (1.3 ppm), long-term stability, and excellent repeatability. The gas sensing mechanism for enhanced sensing performance of nanocomposite is possibly attributed to the formation of p-n heterojunction and the active interaction between WO_3_ nanolamellae and rGO sheets. Besides, the introduction of rGO nanosheets leads to the impurity of synthesized materials, which creates more defects and promotes larger specific area for gas adsorption, outstanding conductivity, and faster carrier transport. The superior gas sensing properties of WO_3_/rGO based gas sensor may contribute to the development of a high-performance ppm-level gas sensor for the online monitoring of dissolved C_2_H_2_ gas in large-scale transformer oil.

## 1. Introduction

Large-scale power transformers are one of the most essential equipment in power system and the stability of the power system will be seriously disturbed when faults occur during the normal operation of power transformers [1]. These faults have the possibility to cause the degradation of power quality, blackouts, and lead to a considerable property loss [2]. Therefore, it is significant to monitoring the operation state of large-scale power transformers and guarantee their proper functioning. During the long-time running of power transformers, incipient thermal or discharge faults might occur at high voltage working condition and cause physical damage to the insulation system inside power transformers which is mainly composed of paper and oil [3,4]. Hence, the C-C and C-H bond may decompose and generate a serious of hydrogen and carbon compound when the insulation system is exposed to extreme heat and discharging [5]. These hydrogen and carbon might react with each other and lead to the formation of various dissolved gases, like hydrogen (H_2_), carbon monoxide (CO), carbon oxide (CO_2_), methane (CH_4_), and acetylene (C_2_H_2_) [6]. Existing faults diagnosis methods, such as IEC ratio method [7], Rogers ratio method [8], are mainly based on the concentrations proportion of different dissolved gases. Therefore, it is necessary to detect concentrations of dissolved gases in power transformer. In addition, the C_2_H_2_ gas is a colorless combustible hydrocarbon with fragrance, which is widely utilized in many industrial fields, like welding, metal cutting, and in dry-cell batteries, etc. However, the explosion limit of C_2_H_2_ gas is 2.3–72.3% (Vol) and it has the danger of explosion under conditions, like heating, shake, and electric spark [9,10]. Thus, it is crucial to detect the concentrations of C_2_H_2_ gas in other industrial fields as well except the C_2_H_2_ detection on power transformer faults monitoring and diagnosis field.

Metal Oxides (MOs), which change their resistance in ambient gases, are promising materials for gas type and concentration detection [11]. Many MOs are appropriate for gas detection, such as CuO, NiO, TiO_3_, ZnO, etc. Among various MOs, tungsten trioxide (WO_3_) is an important n-type semiconductor and it has been considered as one of the outstanding gas sensing materials because of its applicability for reducing and oxidizing gases detection, low cost, and being environmentally friendly [12,13,14]. Furthermore, morphological modulation and doping of WO_3_ has been utilized to enhance the sensing response. Flower-like WO_3_ [15], Pd-doped WO_3_ [16], and Pt-doped WO_3_ [17] have been reported for WO_3_ gas sensing performance promotion. However, drawbacks like high working temperature, poor selectivity still restrain the application of WO_3_ gas sensing materials [18].

To overcome these limitations and improve the gas sensing performance of WO_3_ sensing materials, the hybridization of WO_3_ and reduced graphene oxide (rGO) have been investigated. The strong interaction between WO_3_ and rGO improves sensing properties, because of excellent chemical, electronic, and mechanical properties of rGO. rGO provides more adsorption sites, larger specific areas, and high conductivity to enhance the gas sensing capability. Kaur et al. [19] investigated the influence of temperature on selective detection of hydrogen and acetone based on WO_3_/rGO nanocomposites and found that the introduction of rGO reduced the optimal temperature. Shi et al. [20] investigated H_2_S sensing by employing reduced graphene oxide/hexagonal WO_3_ nanosheet composites. Although, other research groups have devoted plenty of time on the trace H_2_, CO_2_, CO, H_2_S, and CH_4_ gas detection and many promising detection achievements have been obtained. However, there are few studies on C_2_H_2_ gas detection at the ppm level. Our group has reported Au and Pt-doped SnO_2_ nanocomposites that were prepared by hydrothermal method for acetylene detection, which is restrained by the cost [21]. Zhang et al. [22] has reported WO_3_ nanowire and nanorod were used to detect acetylene. However, the concentration of acetylene detected cannot meet requirements for transformer faults diagnosis. The application of WO_3_/rGO nanocomposites prepared through simple hydrothermal method for acetylene detection has not been investigated yet, and it is significant to find such a material for ppm level acetylene detection. Nanocomposites based high performance sensors are cost effective which make it practical for industrial production.

In this work, we investigated gas sensing properties of prepared materials, including the optimal working temperature, dynamic sensing transient, response and recovery time, long-term stability, and repeatability. We employed hydrothermal method to synthesize four contents of WO_3_/rGO nanocomposites (0.5, 1, 2, 4 wt %). Pure WO_3_ and rGO were synthesized as comparison as well. Subsequently, prepared samples were characterized by X-ray diffraction (XRD), Raman spectra, transmission electron microscopy (TEM), X-ray photoelectron spectroscopy (XPS), thermogravimetric analyses (TG), differential scanning calorimetry (DSC), derivative thermogravimetric analysis (DTG), BET analyses, and photoluminescence (PL) emission. Afterwards, ceramic planar gas sensors were fabricated to examine the gas sensing performance to ppm level C_2_H_2_ gas. Ultimately, the gas sensing mechanism was discussed. The main aim of this work is to fabricate a high-performance C_2_H_2_ gas sensor that can operate at low temperature for large power transformer faults monitoring.

## 2. Materials and Methods

### 2.1. Materials

The commercially available extra pure graphite fine powder was purchased from XFNANO Materials Technology Co., Ltd. (Nanjing, China). Sulphuric acid (98%), potassium permanganate (99%), Hydrazine hydrate (80%), ammonia solution (30%), and diallyldimethylammonium chloride (PDDA) solution (60%) were purchased from Aladdin Chemical Reagent Co., Ltd. (Shanghai, China). Sodium tungstate dihydrate (Na_2_WO_4_·2H_2_O) and hydrochloric acid (35–38%) were purchased from Chuandong Chemical Reagent Co., Ltd. (Chongqing, China). All the chemicals were used as received without further purification.

### 2.2. Synthesis of WO_3_/rGO Nanocomposites

Graphite oxide (GO), which is the precursor of rGO, has been prepared from extra pure graphite powder by utilizing the well-known modified Hummer’s method [23]. Four proportions of WO_3_/rGO nanocomposites were synthesized via hydrothermal method by using different amount of GO (0.5, 1, 2, and 4 wt %). Firstly, an appropriate amount of GO was added into 50 mL deionized water that contained 4 mL of PDDA and then ultrasonicated for 1 h to achieve a dispersed suspension. It is worth to be noticed that nanocomposites prepared without use of PDDA result in almost no attachment between WO_3_ nanolamellae and rGO sheets. Next, 2 g of Na_2_WO_4_·2H_2_O was dissolved into the above precursor solution and was kept stirring for 0.5 h. Afterwards, a certain ratio of acid solution (10 mL HCI in 20 mL deionized water) was added dropwise into the above solution under sustaining stirring until the pH value was adjusted to 2.0. Furthermore, 1 mL of hydrazine hydrate was added into the mixed solution. The hydrazine hydrate was used here to reduce GO into rGO. Subsequently, the solution was transferred to 100 mL Teflon-lined, stainless-steel autoclave that was heated at 180 °C for 24 h. The obtained products were collected in culture dishes after centrifugal separation and washing for several times. Ultimately, culture dishes with products were moved to a drying oven for overnight drying at 60 °C.

The pure WO_3_ samples were prepared as a comparison group as well and the preparation process is the same as above steps, except the addition of GO and PDDA.

### 2.3. Characterization Techniques

The microstructure and morphology of prepared samples were analyzed. X-ray diffraction (XRD) data were recorded on Bruker D8 Advance (Bruker, Karlsruhe, Germany) at 40 kV/30 mA with Cu $k_{\alpha }$ radiation (λ = 0.15418 nm). Transmission electron microscopy (TEM) and high-resolution microscopy (HRTEM) were analyzed on JEOL JEM-2000EX (JEOL, Tokyo, Japan). Raman spectra was conducted on HORIBA Jobin Yvon LaRam (HORIBA Jobin Yvon, Paris, France), equipped with a 532 nm wavelength laser and full-range grating. X-ray photoelectron spectroscopy (XPS) was utilized to study surface chemical analysis by Thermo ESCALAB 250Xi spectrometer (Thermo Fisher Scientific, Waltham, MA, USA) with an Al-$k_{\alpha }$ radiation source. TG-DSC-DTG analysis was operated on PerkinElmer TGA-8000 (PerkinElmer, Waltham, MA, USA) in nitrogen atmosphere at a heating rate of 10 °C min^−1^ at the temperature ranging from room temperature to 800 °C. The specific surface area was measured on JWGB JW-BK 100 (JWGB, Beijing, China). Photoluminescence emission spectra (PL) were obtained using 280 nm as an excitation wavelength on PerkinElmer Lamba 45 (PerkinElmer, Waltham, MA, USA).

### 2.4. Fabrication of Planar Gas Sensor

**Figure 1:**
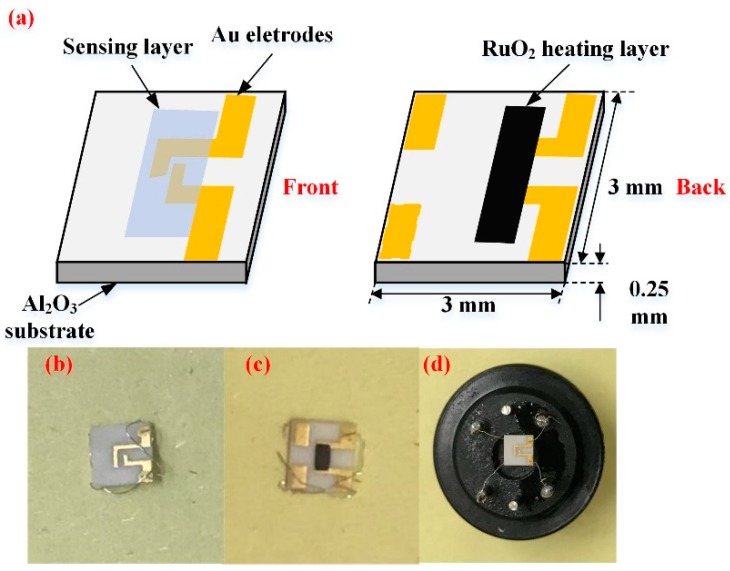
(**a**) Schematic diagram of the tungsten trioxide/reduced graphene oxide (WO_3_/rGO) nanocomposites ceramic planar gas sensor; (**b**) Photographs of the blank sensor (front); (**c**) Photographs of the blank sensor (back); and, (**d**) Photographs of the well-fabricated sensor.

To study the gas sensing effect of synthesized samples, a ceramic planar gas sensor was fabricated. The sensor is mainly composed of fours parts: sensing layer, Au electrode, Al_2_O_3_ substrate, and RuO_2_ heating layer. Figure 1 is the schematic diagram of adoptive planar sensor in this work. Interdigital Au electrode with approximately 300 nm thick foil, 0.2 mm electrode width is manufactured via Chemical Vapor Deposition (CVD) method, and spacing by screen printing technique on 3 × 3 × 0.25 mm (Length × Width × Height) Al_2_O_3_ substrate [24,25]. RuO_2_ layer with approximately 300 nm thick and 0.4 mm^2^ area is deposited on the back side of Al_2_O_3_ substrate as a heater. RuO_2_ heating layer have properties, like low power dissipation, corrosion resistance, and reliable operation [26]. Therefore, it has been used for chemical sensors for many cases.

Four contents of WO_3_/rGO nanocomposites were evenly dispersed in a mixture of distilled water and ethanol (ratio 1:1). Next, the binder solution comprising ethyl cellulose (Aladdin Ltd., Shanghai, China, 90–110 mPa) and α-terpineol (Aladdin Ltd., Shanghai, China, 90%) is added into the mixture. Samples were then transferred to a mortar for a 30 min grind to obtain homogeneous gas sensing test paste. Well-mixed test paste was spin-coated on Al_2_O_3_ substrate and interdigitated electrodes at 3000 rpm for 30 s to acquire a dense and uniform coating [27]. Subsequently, planar sensors that were coated with corresponding gas sensing materials were aged at 150 °C for 1 h and then annealed at 300 °C for 2 h in an oven for binder removal. Sensors fabricated from pure rGO, pure WO_3_ and WO_3_ with 0.5, 1, 2, and 4 wt % GO were labelled as W0G, WG0, WG0.5, WG1, WG2, and WG4, respectively.

### 2.5. Gas Sensing Measurements

**Figure 2:**
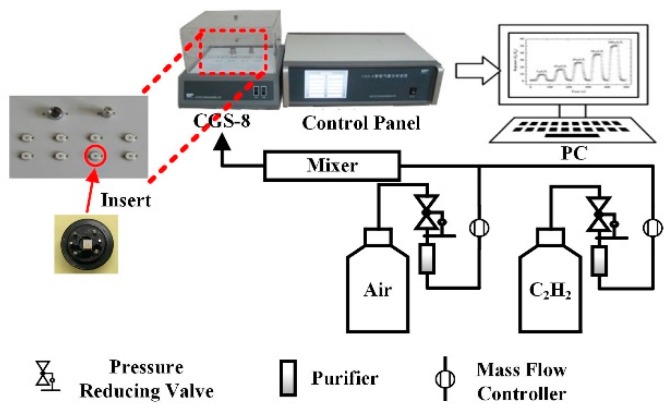
Gas sensing test system.

The gas sensing effect of prepared ceramic planar gas sensors for C_2_H_2_ were investigated by our gas sensing test analysis system. The total test system that is shown in Figure 2 is made up of a PC, an automatic gas distribution system, a CGS-8 gas sensing characterization analysis system (Elite Technology Co., Ltd., Beijing, China), and a control panel. This analysis system is convenient for adjusting experimental condition and obtaining experimental parameters (working voltage, operating temperature, ambient condition, ohm characteristics, etc.).

Steps of gas sensing test are described below. First, the ceramic planar gas sensors coated with prepared samples were welded on experimental interfaces, which is shown in Figure 1d. Then, well-welded gas sensors were connected to CGS-8 gas sensing characterization analysis system. Subsequently, the operating temperature of sensors was set and air as a carrier gas was injected into the experimental chamber at a constant flux. Next, C_2_H_2_ gas was injected when the resistance of planar gas sensors remained stable. After the response data was obtained, air was injected into the chamber again to carry the C_2_H_2_ gas out. The concentration of C_2_H_2_ gas was controlled by the mass flow controllers (MFCs) with the following equation:

(1) $${\mathrm{Gas}}_{\mathrm{concentration}}(\mathrm{ppm})=\frac{{\mathrm{Flux}}_{}\;{\mathrm{rate}}_{C_{2}H_{2}}}{{\mathrm{Flux}}_{}\;{\mathrm{rate}}_{\mathrm{air}}+{\mathrm{Flux}}_{}\;{\mathrm{rate}}_{C_{2}H_{2}}}\;$$

Sensors were tested at temperature ranging from 50 °C to 400 °C for various concentrations of C_2_H_2_ gas. The response of sensors was defined as the ratio S = Ra/Rg, where Ra is the resistance of sensors in air and Rg is the resistance of sensors in C_2_H_2_ gas at the corresponding concentration. The response time is defined as the time that is taken for sensors to reach 90% of the total resistance change while the recovery time denotes the time needed that 90% of baseline resistance is recovered. The environmental temperature (30 °C) and humidity (25% RH) were kept the constant during the whole experimental process.

## 3. Results and Discussion

### 3.1. Structural Characterization

**Figure 3:**
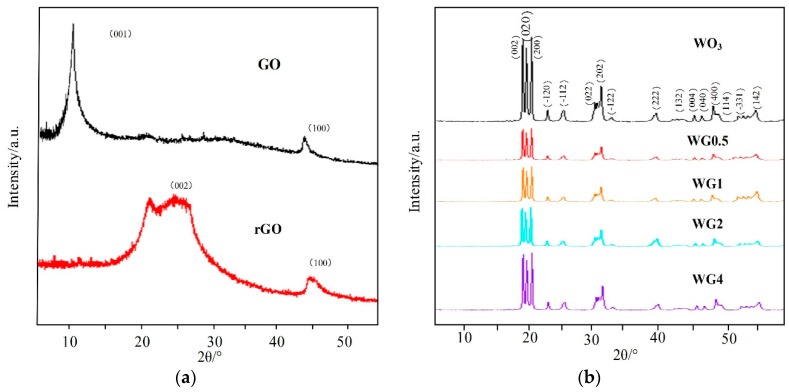
(**a**) A X-ray diffraction (XRD) patterns of graphite oxide (GO) and rGO; (**b**) XRD patterns of WO_3_ and different concentrations of WO_3_/rGO nanocomposites.

The morphological and structure characterization of prepared sample were detected before gas sensing performance measurement. The crystal structure and phase purity of prepared samples were characterized by XRD. Figure 3a,b illustrates the XRD patterns of GO, rGO, pure WO_3_, and four contents of WO_3_/rGO. In Figure 3a, a strong peak at 2θ = 10.90° in XRD pattern of GO, which is related to (001) crystal plane was observed. This peak vanishes when GO are reduced into rGO and replaced by a new peak at 2θ = 24.50° related to (002) crystal plane of rGO (JCPDS-41-1445) [28,29]. In Figure 3b, the XRD pattern of the pure WO_3_ shows three strong peaks ranging from 22° to 25°, which correspond to crystal planes (200), (020), and (002), and these three planes are consistent with the monoclinic phase of WO_3_ nanostructure (JCPDS-43-1035) [30]. Interestingly, in WO_3_/rGO nanocomposites, no peak related to GO or rGO appears in XRD patterns and the intensity of four contents of WO_3_/rGO almost have no difference. This is possibly due to the low content and proper exfoliation of rGO sheets [31,32].

**Figure 4:**
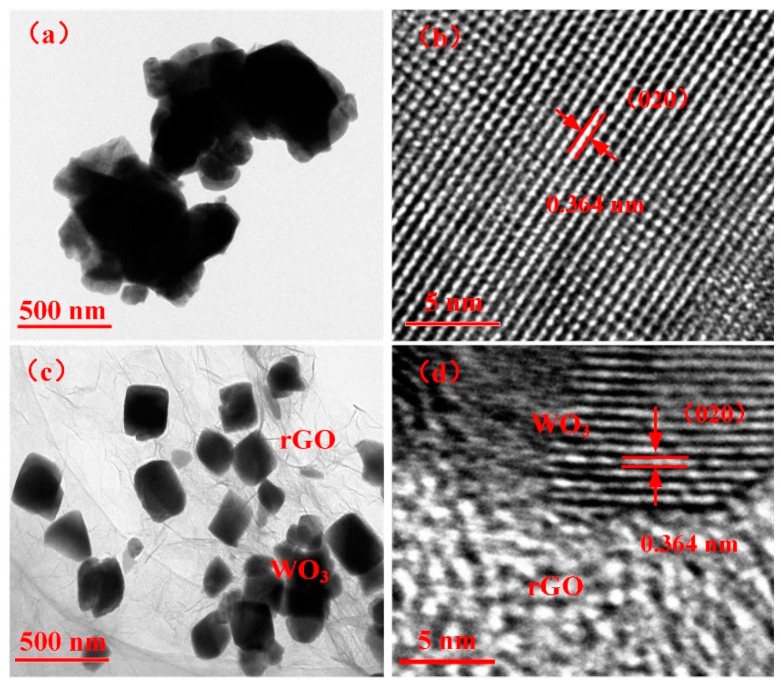
(**a**) Transmission electron microscopy (TEM) image of pure WO_3_ nanolamellae; (**b**) high-resolution TEM (HRTEM) image of WO_3_ nanolamellae; (**c**) TEM image of WG1 nanocomposites; and, (**d**) HRTEM image of WG1 nanocomposites.

The TEM and high-resolution TEM (HRTEM) micrographs of WO_3_ and WG1 nanocomposites are displayed in Figure 4. Figure 4a shows the TEM micrograph of WO_3_ and it demonstrates the nanolamellae-like micrograph of the pure WO_3_ sample. The TEM micrograph of WG1 nanocomposites is exhibited in Figure 4c. The micrograph of WO_3_/rGO proves the excellent quality of prepared nanocomposites because WO_3_ nanolamellae are radicated on crumpled rGO sheets. Besides, obvious fold boundary is observed, which indicates the strong interaction between WO_3_ nanolamellae and rGO sheets. The HRTEM image of WO_3_ nanolamellae and WO_3_/rGO nanocomposites are shown in Figure 4b,d, which demonstrates a homogeneous neighboring fringe spacing of WO_3_ is 0.364 nm and it corresponds to the (020) plane of monoclinic WO_3_.

**Figure 5:**
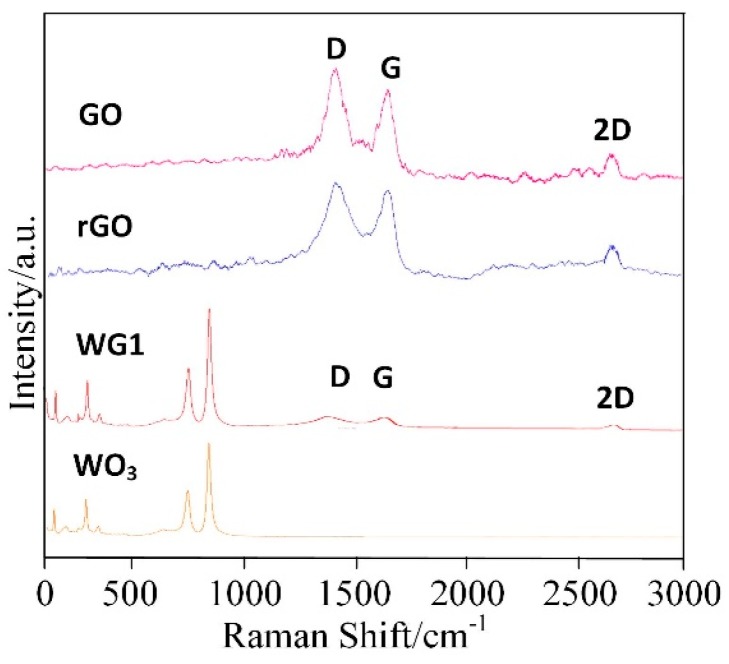
Raman spectra of rGO, GO, WG1, and WO_3_ nanocomposites.

Figure 5 shows the Raman spectra of prepared GO, rGO, pure WO_3_ and WG1 nanocomposites. Raman spectra is a non-destructive and fast method to characterize WO_3_ and WO_3_ derivatives [33]. In this work, a 532 nm wavelength laser as an excitation was utilized. The Raman spectroscopy of GO shows three remarkable peaks located at 1342 cm^−1^, 1600 cm^−1^, and 2698 cm^−1^. These three peaks are the well-known disorder-induced (D) band, tangential (G) band, and second-order (2D) band of graphitic materials, respectively [34,35]. After the ultrasonic exfoliation and hydrothermal method, GO was successfully reduced and a tiny shifting of D and G band to lower wavenumbers was observed in rGO and WG1 nanocomposites. Besides, the intensity ratio of the D and G bands (ID/IG) was calculated. The intensity ratio of WG1 and rGO increased compared with GO which also proved GO has been reduced to rGO. The increase of intensity ratio value in WG1 nanocomposite and rGO possibly attributed to the presence of plenty of defects and disorder [36]. Generally, the higher the intensity ratio is, the more defects and disorder are generated in carbon materials [35]. These defects promote the adsorption of oxygen and gas molecules which enhance the gas sensing response [37]. The characteristic bands of WO_3_ were also detected in WG1 nanocomposites sample. Two strong Raman bands appeared at about 812 cm^−1^ and 707 cm^−1^ and this two bands are allocated to stretching vibrational modes of tungsten oxygen bond [38]. Raman bands at about 272 cm^−1^ and 83 cm^−1^ are allocated to bending vibrational modes of tungsten oxygen bond [39]. When compared with rGO, the D band is slightly blue-shift by 8 cm^−1^ in the WG1 nanocomposite, while the G band shows a red-shift of 3 cm^−1^. These shifts in Raman peak could be attributed to the interaction between WO_3_ and rGO again. Xu et al. [40] have reported similar experimental results when exploring the Raman peak shift of ZnO/graphene composites. Besides, the tiny bands shifting of D and G band observed in WG1 could be possibly explained by tensile stress at the interface of WO_3_ and rGO as well. Similarly, experimental phenomenon has been observed by Lo et al. [41].

**Figure 6:**
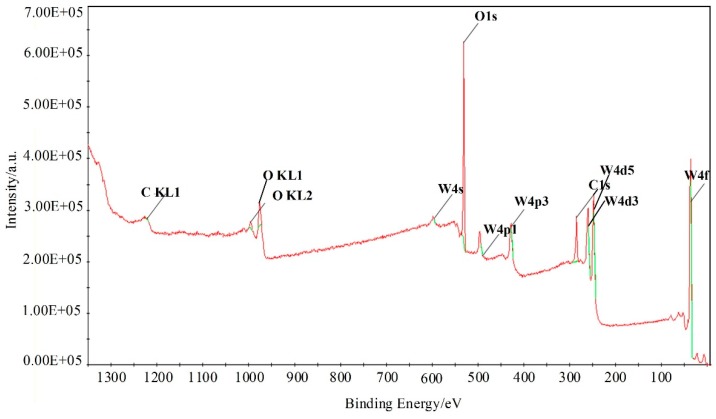
XPS (X-ray photoelectron spectroscopy) spectra of WG1 nanocomposites.

**Figure 7:**
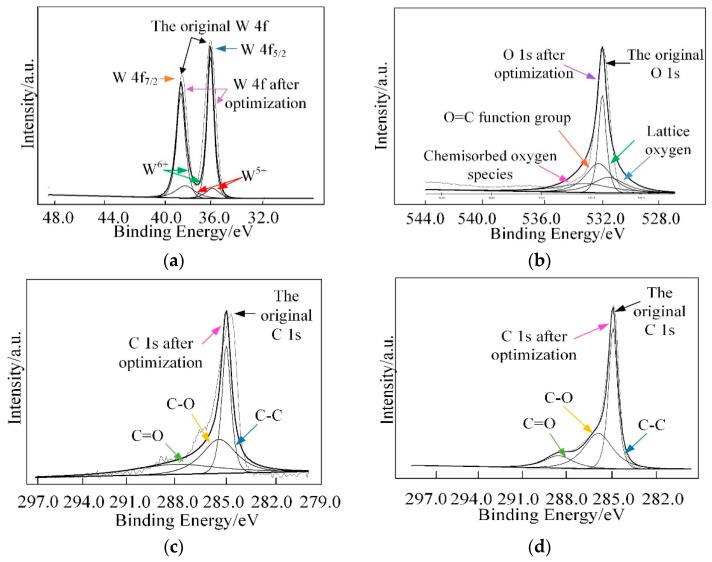
(**a**) High-resolution XPS spectra in the vicinity of the W 4f peaks; (**b**) O 1s peak; (**c**) C 1s peak of WG1; and, (**d**) C 1s peaks of rGO.

Furthermore, X-ray photoelectron spectroscopy (XPS) analysis was employed to investigate the surface compositions and chemical states of WG1 nanocomposites, which is shown in Figure 6. As Figure 6 depicted, the peaks of element W, C, and O were present in XPS. Two peaks with binding energy of 36.00 and 38.16 eV are corresponding to W 4f_5/2_ and W4 f_7/2_, respectively. These two peaks indicate the existence of W^6+^. Another two lower intensity peaks at 35.90 and 37.95 eV that are related to W^5+^ are observed simultaneously. Three peaks at 529.8, 530.7, and 531.7 eV were obtained after deconvolution of the O 1s peak. Among these three peaks, the 530.6 eV and 531.1 eV peaks are attributed to oxidized metal ions in the lattice of WO_3_, while 531.4 eV peak corresponds to O=C surface groups in GO and the oxygen in WO_3_. The 532.5 eV peak originated from the oxygen chemisorbed on the surface of prepared nanocomposites. The C 1s peak of WG1 can be separated into three peaks by deconvolution at 285.0 285.5, and 288.5 eV, which are corresponding to the binding energy of C-C, C-O, and C=O, respectively. To ensure a successful reduction reaction of GO in prepared WG1 sample, the C 1s peak of rGO was shown in Figure 7d. Three peaks located at 284.8, 285.8, and 288.5 eV were demonstrated after deconvolution on the original C 1s peak of rGO. The binding energy of C-C, C-O, and C=O in C 1s peak of rGO were in good agreement with the outcomes of C 1s peak in prepared WG1 sample.

**Figure 8:**
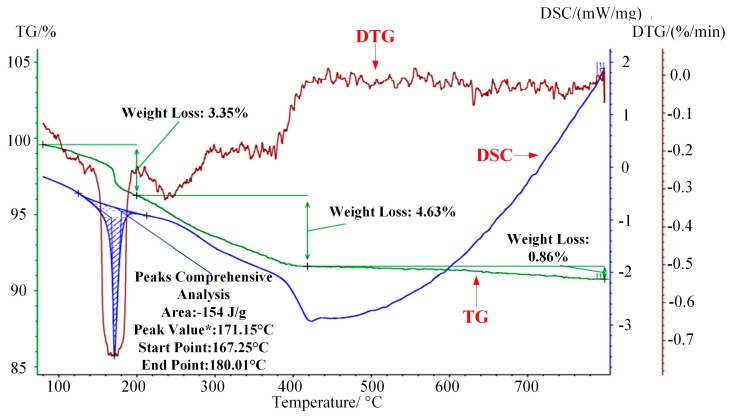
Thermogravimetric analyses-derivative thermogravimetric analysis-differential scanning calorimetry (TG-DSC-DTG) curves of prepared WG1 sample.

The combustion characteristics of WG1 nanocomposites were investigated by utilizing thermogravimetric analysis (TG), differential scanning calorimetry (DSC), and derivative thermogravimetric analysis (DTG). Experimental settings were described, as follows: temperature scanning rate: 10 °C/min; temperature range 80–800 °C and protective atmosphere: N_2_. As the Figure 8 depicted, the first 3.35% mass loss at around 170 °C, which was accompanied by a sharp DSC peak, was due to the elimination of adsorbed water molecular and the DTG curves showed that a rapid weight loss happened here [42]. Subsequently, a 4.63% weight loss occurred ranging from 200 to 420 °C because of the elimination of oxygen-containing functional groups. CO, CO_2_, and steam generated along with the pyrolysis of oxygen-containing functional groups, which was along with a progressively decreasing DSC curve [43]. Further weight loss occurred with the pyrolysis of carbon skeleton when temperature was above 450 °C, which was also proved by the curve trend of DSC [44]. It can be concluded that prepared WG1 nanocomposites have good thermal stability at high temperature and the microstructure of WG1 nanocomposites remain stable under 300 °C without much weight loss. The combustion characteristics of WG1 results from the strong interaction between WO_3_ nanolamellae and rGO sheets.

BET was employed to investigate the specific area of prepared samples. Specific area is a significant factor for the application of synthesized gas sensing materials. Larger specific area can supply more active sites for gas adsorption and enhance the gas sensing response [45]. The BET specific area of WO_3_ nanolamellae and WG1 nanocomposites were examined. From the measurement outcomes, it showed the BET specific area of WG1 nanocomposites was 25.32 m^2^/g and much larger than the specific area of pure WO_3_ nanolamellae, which was 6.3 m^2^/g. Based on the BET analysis results, it proved that the introduction of rGO enlarge the specific areas of nanocomposites as compared to pure WO_3_ nanolamellae. As a result, interactions between the detection gas and gas sensing materials are improved and sensor response is enhanced. 

**Figure 9:**
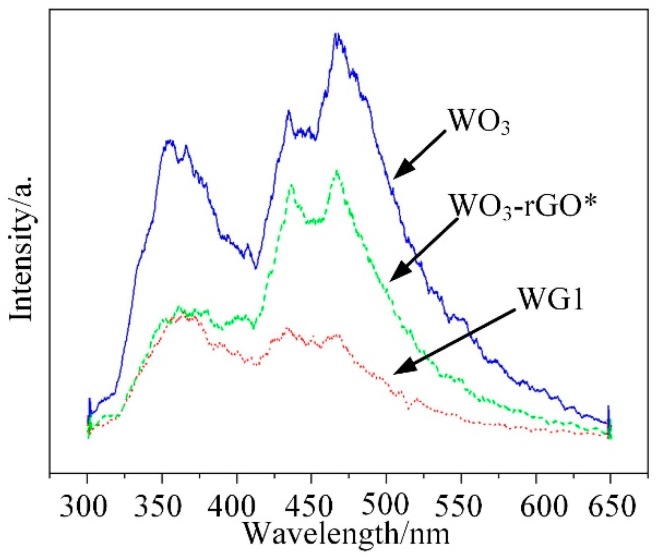
Photoluminescence (PL) emission spectra for pure WO_3_ and WG1 nanocomposite. WO_3_-rGO*: the mechanical mixture of WO_3_ and rGO (1 wt % GO).

Photoluminescence (PL) emission results from the combination of electrons and holes and it can be employed to analyze the impurity and defects distribution of synthesized materials [46]. In the emission spectra, low emission intensity represents a low combination rate of electron hole pairs. Besides, it also supplies the information about the energy states of conduction band and valence band. In this work, PL measurements of WO_3_, WG1, and WO_3_-rGO (mechanical mixture of WO_3_ and 1%wt GO) nanocomposites were operated at room temperature (30 °C) and the spectra was recorded under the excitation of 280 nm wavelength laser. Three highlighted peaks at 356, 434, and 467 nm were presented in Figure 9, which represented the PL spectra of WO_3_. Among these three peaks, the peak at 356 nm is caused by the near-band edge transition, while the peak at 434 nm is related to the band-to-band transition [47,48]. Besides, the 467 nm band is corresponding to existence of oxygen vacancies [49]. When compared to the PL emission results of WO_3_, the PL emission band peak of WG1 nanocomposites is notably quenched by rGO [50]. Similar experimental outcomes have been reported. Zhou et al. [51] have reported metal oxide/rGO nanocomposites had significant PL intensity decreasing as compared to same metal oxide nanocomposites without rGO. Yu et al. [52] observed that Sn_3_O_4_/rGO heterostructure shows much lower intensity of the emission peak when compared to Sn_3_O_4_ nanosheets in the same wavelength range. This outcome demonstrated that the rGO sheets serve as electrons receivers for the WO_3_ nanolamellae anchoring on them and weak the radiative recombination of electrons and holes. Because rGO sheets serve as electrons receivers, an additional pathway for electrons transfer from the conduction band of excited WO_3_ to conducting rGO sheets is formed. Consequently, electrons flow across the WO_3_-rGO interface was markedly enhanced by the electrons that transfer from WO_3_ nanolamellae to rGO sheets, and the rGO also has a good transport capability, which enhances the performance of prepared nanocomposites [53]. Besides, the introduction of rGO sheets in WO_3_ nanolamellae prevents the recombination of electrons holes pairs to some degree. Hence, it reduces oxygen to create superoxide radical species with proficient oxidation ability for gas reaction [54]. Ultimately, the PL measurement was operated on the WO_3_-rGO sample, as well to verify the interaction between WO_3_ nanolamellae and rGO nanosheets. It is interesting to notice that the PL emission intensity of WO_3_-rGO is between those of WO_3_ and WG1. Moreover, the intensity of WO_3_-rGO decreases lightly, which is possibly attributed to the poor contact of mechanical mixing WO_3_ and rGO. Chen et al. [55] has reported similar PL emission intensity outcomes when studying the PL properties of pure g-Nis, g-C_3_N_4_/NiS-1.5%, and mechanical mixture of g-C_3_N_4_ and NiS.

### 3.2. C_2_H_2_ Gas Sensing Properties

**Figure 10:**
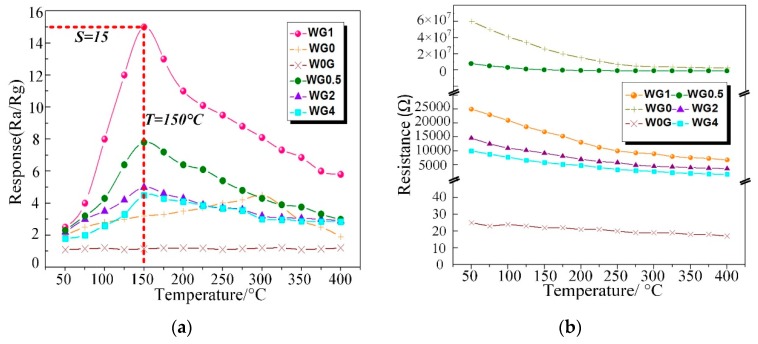
(**a**) Response of W0G, WG0, WG0.5, WG1, WG2, and WG4 to 50 ppm C_2_H_2_ at different operating temperature; (**b**) Resistance of W0G, WG0, WG0.5, WG1, WG2, and WG4 at different operating temperature in air.

C_2_H_2_ gas is one of the dominant fault characteristic gases dissolved in power transformer oil and the power station staff can recognize the fault type based on the amount of C_2_H_2_ gas generated [56]. Therefore, the performance of our fabricated WO_3_/rGO nanocomposites sensors need to be tested to investigate the sensing properties to C_2_H_2_ gas. To obtain the optimal working temperature, W0G, WG0, WG0.5, WG1, WG2, and WG4 were exposed to 50 ppm C_2_H_2_ gas in the experimental chamber at the temperature ranging from 50 °C to 400 °C with the interval of 50 °C. Figure 10a shows the relationship between sensing response of different sensors and working temperature. The results that were obtained distinctly exhibit that the sensors response of WG0, WG0.5, WG1, WG2, and WG4 increase firstly and decrease then with the temperature rise. However, no clear fluctuation is observed for W0G curve in Figure 10a and W0G shows a weak response to C_2_H_2_ gas. Besides, the resistance curve (resistance of W0G, WG0, WG0.5, WG1, WG2, and WG4 in the air versus temperature) was given in Figure 10b. It can be clearly observed that WG0.5, WG1, WG2, and WG4 show an evidently enhancement in electrical conductivity as compared to WG0. Furthermore, the resistance of WG0.5, WG1, WG2, and WG4 decreases dramatically with the increase of GO contents.

Besides, the optimal working temperature of WG0.5, WG1, WG2, and WG4 (about 150 °C) is lower than WG0 (about 300 °C) and the sensor response of WG1 is much higher than W0G, WG0, WG0.5, WG2, and WG4 at their best working temperature. The possible explanation for this experimental phenomenon is that sensors have a small chemical activation at low working temperature and a potential barrier formed by the chemisorbed oxygen ions on the gas sensing materials surface prevents the reaction between C_2_H_2_ gas and materials. The activity of prepared materials is activated progressively with the rising of temperature, and therefore, sensors response have an enhancement. However, the adsorbed gas molecular may escape before reaction with the sustained rise of temperature, which leads to a response drop. Besides, the elimination of oxygen-containing functional groups may lead to a response decrease at high temperature as well. It can be concluded that the optimal working temperature of sensors with hybridization of WO_3_ and rGO can be efficiently reduced as compared to pure WO_3_ or rGO sensors and the gas response is highly improved with the hybridization at optimum working temperature.

**Figure 11:**
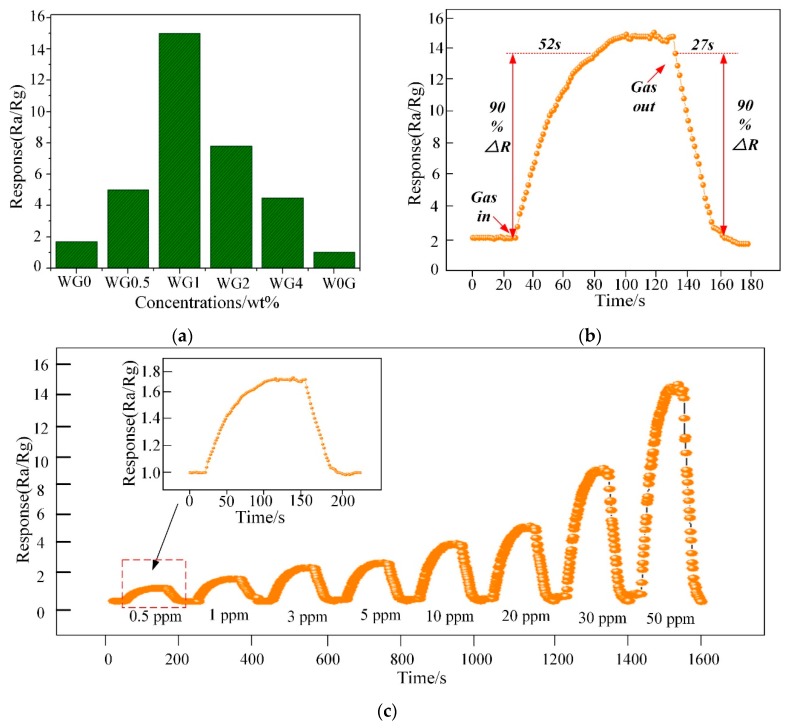
(**a**) Sensor response variation for 50 ppm C_2_H_2_ at 150 °C; (**b**) Dynamic sensing transient of WG1 senor to 50 ppm C_2_H_2_ gas; and, (**c**) Response under different gas concentrations from 0.5 ppm to 50 ppm for C_2_H_2_ of WG1 at 150 °C.

Figure 11a illustrates the response of WG0, W0G, WG0.5, WG1, WG2, and WG4 to 50 ppm C_2_H_2_ gas at 150 °C. It can be observed that WG1 has the optimal response Ra/Rg = 15 to the C_2_H_2_ gas and hence the WG1 was selected for further characterizations. The response-recovery curve of WG1 to 50 ppm C_2_H_2_ gas at the best working temperature is demonstrated in Figure 11b and the WG1 has the best response with minimum response (52s) and recovery time (27s). Furthermore, WG1 was exposed to different concentrations of C_2_H_2_ gas ranging from 0.5 ppm to 50 ppm to investigate the dynamic response-recovery curve. As Figure 11c depicted, it is obvious to be noticed that the response of WG1 increased when C_2_H_2_ was injected into experimental chamber and the response decreased to the base line value when C_2_H_2_ was carried out via air.

The alarm value of C_2_H_2_ gas concentration in operative 220 kV and below power transformer is 5 ppm. Therefore, the detection of low concentration of C_2_H_2_ gas is highly important. The WG1 nanocomposites sensor can detect even 1.3 ppm efficiently, and it shows practicability in power transformer fault characteristic gas detection and fault type estimation.

**Figure 12:**
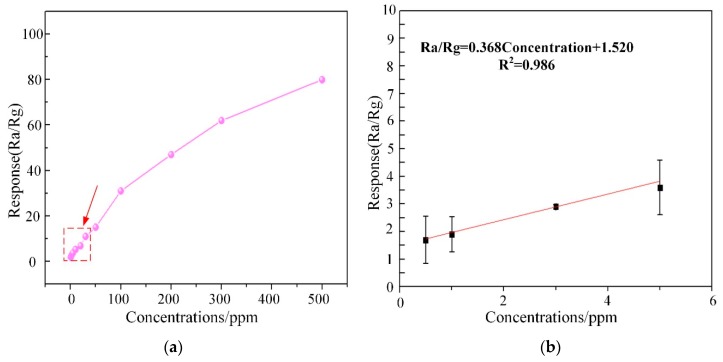
(**a**) Corresponding response variations of the WG1 sensor as a function of C_2_H_2_ gas concentrations at 150 °C; and, (**b**) Linear relationship between response and concentrations at low concentrations.

Subsequently, the response curve of WG1 gas sensor to multi-concentrations of C_2_H_2_ gas ranging from 0.5 ppm to 400 ppm at the best working temperature was depicted in Figure 12a. The response of WG1 increased progressively at low C_2_H_2_ gas concentration and it got saturation at high concentration. The potential explanation is that no more adsorption sites on the WG1 surface can be supplied for C_2_H_2_ gas and the reaction between WG1 and C_2_H_2_ gas molecular has reached a dynamic equilibrium. Besides, Figure 12b, which is the enlarged view of box part in Figure 12a, shows that the gas concentration-response curve of WG1 has a good linearity when the C_2_H_2_ concentration is below 10 ppm. The linear relationship between concentrations and response can be summarized as one equation, exhibited below:

(2)  y = 0.368x + 1.520 

In this work, we defined the lowest detection limitation that can be reached was the lowest concentrations when the response reached 2. Hence, it can be calculated that the lowest detection limitation was 1.3 ppm when the response went to 2.

**Figure 13:**
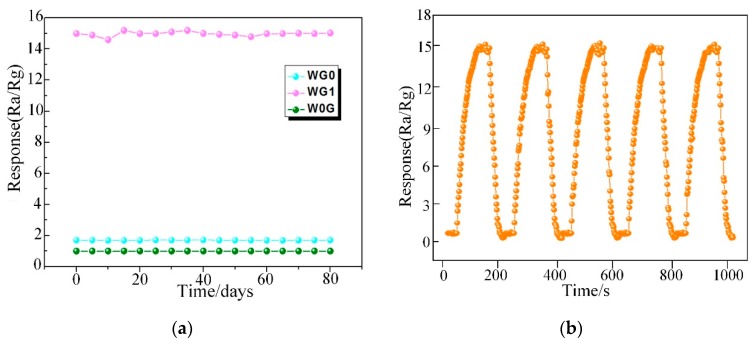
(**a**) The stability of W0G, WG0, and WG1 to 50 ppm C_2_H_2_ gas at 150°C; and, (**b**) The repeatability of WG1 gas sensor to 50 ppm C_2_H_2_ gas at 150°C.

Ultimately, the long term stability of WG0, WG1, and W0G gas sensors were investigated and it is significant for practical application. In this work, W0G, WG0, and WG1 gas sensors were exposed towards 50 ppm C_2_H_2_ gas for 80 days with an interval of five days and the time-response curve was recorded. The outcomes in Figure 13a demonstrate a good long-term stability of WG1 gas sensor. Besides, the repeatability of WG1 gas sensor was tested towards 50 ppm C_2_H_2_ gas for five cycles and the performance of WG1 gas sensor in Figure 13b exhibited a good repeatability during the five cycles.

A lot of previous work has been done by other researchers to study the gas sensing properties of their fabricated gas sensors towards C_2_H_2_ gas. A comparison between our WG1 composites gas sensors and reported gas sensor is given in Table 1.

### 3.3. Gas Sensing Mechanism

The WG1 gas sensor demonstrated an excellent performance on 50 ppm C_2_H_2_ gas detection at 150 °C based on experimental results. Besides, WG0, WG0.5, WG2, and WG4 gas sensors showed the detectability towards 50 ppm C_2_H_2_ gas at 150 °C, which were about 3.2, 7.8, 5.1, and 4.5, respectively. Furthermore, the W0G sensor showed a very low detection capability to C_2_H_2_ gas. Here, the gas sensing mechanism of fabricated gas sensing materials is discussed, as follows.

**Figure 14:**
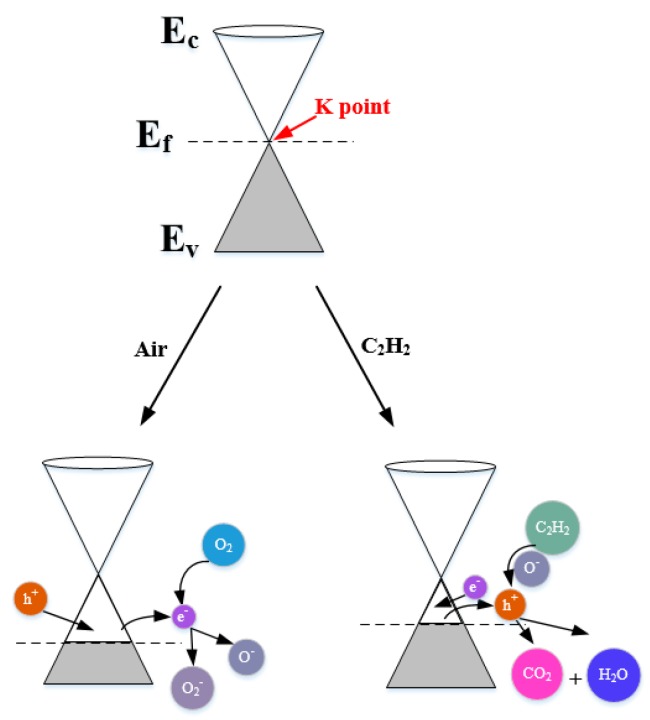
Energy band models for C_2_H_2_ gas sensing mechanisms of pure rGO nanosheets.

Firstly, the experimental outcomes showed that W0G gas sensor showed a p-type gas sensing behavior with very low response towards C_2_H_2_ gas. The p-type C_2_H_2_ gas sensing mechanism can be explained via band diagram, which is illustrated in Figure 14.

It is well-known that rGO has a semi-metallic energy band structure, which means that the conduction band (E_c_) and valence band (E_v_) of rGO meet at the Dirac point K (E_f_) near the edge of Brillouin zone and valence band is full of valence electrons [62,63]. The Fermi level passes the K point under the natural state. When W0G gas sensor is exposed to air at a working temperature, the oxygen molecules adsorbed on the surface of rGO will grasp electrons from the conduction band to form oxygen species (O_2_^−^ and O^−^) and an amount of holes, namely, positive charge carriers are generated at the same time [64]. Therefore, the W0G exhibits a p-type property with Fermi level below the K point. When W0G is exposed to C_2_H_2_ gas, C_2_H_2_ gas will interact with adsorbed oxygen species, releasing electrons back to the surface of rGO nanosheets, and results in a decrease of holes and rise of sensors resistance. However, the resistance increase in p-type rGO is tiny, because the charge carriers that are generated by gas reaction is much less than charger carriers in rGO sheets. So, W0G gas sensor has a very poor performance on C_2_H_2_ detection.

In case of the WG0 gas sensor, the oxygen molecular will extract electrons from the conduction band of WO_3_ to form the adsorbed oxygen ions which contributes to the formation of depletion region on the surface of WO_3_ with a depletion width (W_d_) and barrier height (H_b_). When electrons are extracted from the conduction band of WO_3_, electrons will pass two adjacent depletion layers and a neutral region. This electrons transfer is mainly based on the thermionic conduction mechanism and only electrons with high-energy can be motivated to move across energy barriers. According to the thermionic conduction mechanism, the electrons mobility will increase exponentially with the decreasing of barrier height [65]. Hence, the specific resistance will decline exponentially with the decreasing of barrier height. When pure WO_3_ is exposed to C_2_H_2_ gas, the adsorbed oxygen ions react with the C_2_H_2_ molecules and the trapped electrons will be released back to the surface of WO_3_ and the barrier height will be reduced, which leads to a decline of resistivity. The possible reaction process [66] is given, as follows:

(3) $$\;O_{2}\left(\mathrm{ads}\right){+2e}^{-}↔{2O}^{-}\left(\mathrm{ads}\right)\;$$

(4) $$\;C_{2}H_{2}\left(\mathrm{gas}\right){\;+\;O}^{-}\left(\mathrm{ads}\right)\to {\mathrm{CO}}_{2}{\;+\;H}_{2}{O\;+\;e}^{-}\;$$

**Figure 15:**
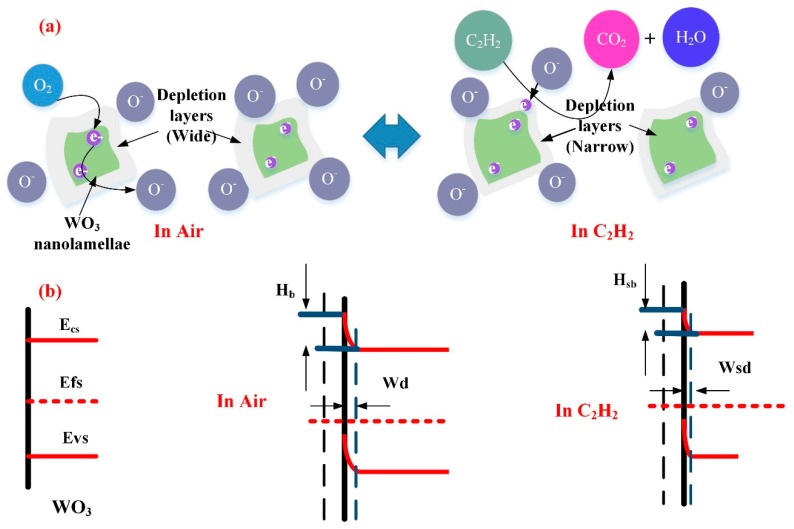
(**a**) Representative physical models for C_2_H_2_ gas sensing mechanism of WG0; and, (**b**) Energy band models for C_2_H_2_ gas sensing mechanisms of WG0.

When electrons move across different WO_3_ nanoparticles, the width of depletion region (W_sd_) and barrier height (H_sb_) are influenced by the interaction between adsorbed oxygen molecular ions and target gases. Hence, by monitoring the change of depletion region width and barrier height, the sensing response of WO_3_ can be measured. Figure 15a,b shows the representative physical models and energy band models for C_2_H_2_ gas sensing mechanisms.

**Figure 16:**
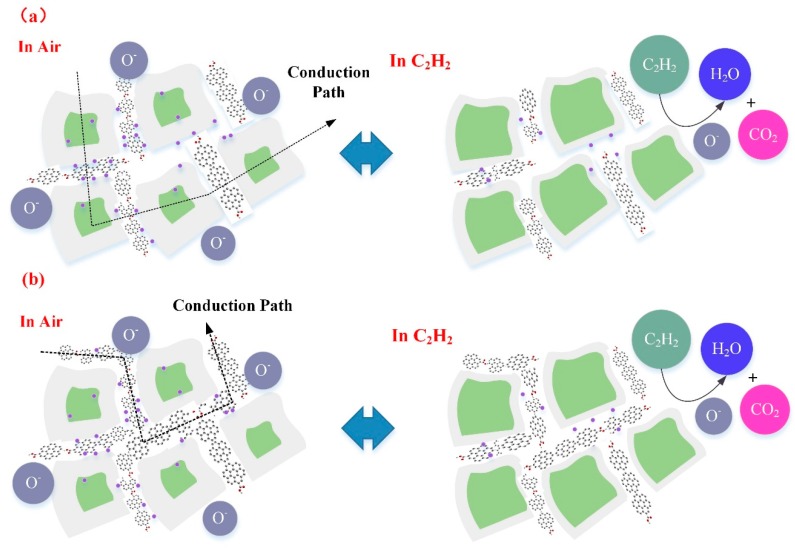
(**a**) Representative physical models for C_2_H_2_ gas sensing mechanism of WG1; and, (**b**) Representative physical models for C_2_H_2_ gas sensing mechanism of WG2 and WG4.

For WG1 gas sensor, the formation of p-n heterojunctions and active interaction between WO_3_ and rGO are the potential reasons for its high performance to C_2_H_2_ gas detection. rGO sheets thata re dispersed discretely among the WO_3_ nanolamellae and p-n heterojunctions are formed between WO_3_ nanolameallae and rGO sheets. Before the adsorption of oxygen molecules ions in air, heterojunctions are ohmic, since the work function of WO_3_ is smaller than rGO. Hence, electrons in WO_3_ will be transferred to rGO and accumulate on the surface of rGO sheets. When the WG1 is exposed in air, the depletion region will form with a barrier height. When comparing the depletion region width and barrier height of WG1 with pure WO_3_, the magnitudes of depletion region width and barrier height of WG1 is larger than pure WO_3_ since the less electrons are acquired from WO_3_. Figure 16a illustrates the physical models for C_2_H_2_ gas sensing mechanisms of WG1.

When WG1 gas sensor is exposed to C_2_H_2_ gas, the C_2_H_2_ gas molecules are strongly adsorbed on the interface of WO_3_ nanolamellae and rGO nanosheets because of highly active sites (oxygen functional groups, vacancies, etc.), which has been verified by the BET analysis as well. The enhanced C_2_H_2_ gas adsorption at the interface will lead an extensive decreasing in the width of depletion region and barrier height. Therefore, the response of WG1, which is the ratio of the resistance in the air and the resistance after C_2_H_2_ reaction, is highly enhanced by two aspects: larger resistance in air and smaller resistance in C_2_H_2_ gas. Additionally, the rGO can act as spacers and prevent the aggregation of WO_3_ nanolamellae. So that, the efficient adsorption and desorption processes can occur. Besides, the introduction of rGO sheets increase the conduction of prepared gas sensing materials, which leads to a faster response and recovery time. All of these factors make the high performance of WG1 gas sensor possible.

For the WG2 and WG4 gas sensor, the response towards C_2_H_2_ gas is smaller than WG1. The reasons from the gas sensing mechanism aspects are discussed, as follows. At a relative high concentration of GO, the rGO sheets homogeneous distributed among the WO_3_ nanolamellae and connected themselves. Because of the connection of rGO sheets, electrons can be transferred easily through the rGO sheets channel and results in a resistance decreasing in air. The width of depletion layers and barrier height will become small as well when C_2_H_2_ gas reacts with adsorbed oxygen molecules ions and contributes to a resistance decline. However, with the increase concentration of GO, the conduction of electrons is partially through the rGO sheets and rGO sheets has a p-type gas sensing property. Therefore, a smaller decrease of resistance can be observed as compared to the WG1 gas sensor. Figure 16b illustrates the physical models for C_2_H_2_ gas sensing mechanisms of WG1.

## 4. Conclusions

In this work, high-performance WO_3_/rGO nanocomposites that were prepared by hydrothermal method were systematically investigated for C_2_H_2_ gas detection. Characterizations by XRD, TEM, Raman spectroscopy, XPS, TG-DTG-DSC, and PL have confirmed that WO_3_ nanolamellae evenly anchored on the high-quality rGO sheets. Gas sensing results revealed that 1%wt GO contents exhibited a high response (15.0–50 ppm), an excellent dynamic response (0.5–50 ppm), a low detection limitation (1.3 ppm), fast response-recovery time (52 s, 27 s), long-term stability, and outstanding repeatability at a relative low optimal working temperature (150 °C). The potential gas sensing mechanism corresponding to different ratio of WO_3_ and rGO were proposed based on the grain boundary theory, formation of p-n heterojunctions, and interaction of WO_3_ nanolamellae and rGO sheets. Therefore, the hydrothermal method WO_3_/rGO nanocomposites sensors are promising high-performance candidates for dissolved C_2_H_2_ gas detection in oil-immersed at ppm-level.

## Figures and Tables

**Table 1:** Comparison between our work and other reported work on C_2_H_2_ gas detection.

Ref	Materials	Temperature/°C	Measuring Range/ppm	Response	τ_Response_/s	τ_Recovery_/s
[22]	WO_3_ nanorod	300	≥200	35 (200 ppm)	10	9
[22]	WO_3_ nanowire	300	≥200	60 (200 ppm)	6	7
[57]	ZnO NPs ^1^	>300	30–1000	6.4 (100 ppm)	>2 min	>3 min
[58]	Ag-ZnO NPs-rGO	150	1–1000	21.2 (100 ppm)	25	80
[57]	ZnO Hrc ^2^	>300	30–1000	11.2 (100 ppm)	>2 min	>6 min
[58]	Ag-ZnO Hrc-rGO	200	3–1000	12.3 (100 ppm)	43	100
[59]	Pure SnO_2_	300	200–10,000	6.3 (10,000 ppm)	34	>10 min
[60]	Pt/ZnO	300	5–10,000	1.1 (50 ppm)	N/A	N/A
[61]	Ag-ZnO Hrc	200	1–1000	57 (50 ppm)	N/A	N/A
Our	WG1	150	1–500	15 (50 ppm)	52	21

^1^ Nps: Nanoparticles, ^2^ Hrc: Hierarchical.

## References

[1] Wang Y.Y., Gong S.L., Grzybowski S. (2011). Reliability evaluation method for oil-paper insulation in power transformers. Energies.

[2] Illias H.A., Liang W.Z. (2018). Identification of transformer fault based on dissolved gas analysis using hybrid support vector machine-modified evolutionary particle swarm optimisation. PLoS ONE.

[3] Wei C., Tang W., Wu Q. (2014). Dissolved gas analysis method based on novel feature prioritisation and support vector machine. IET Electr. Power Appl..

[4] Lundgaard L.E., Hansen W., Linhjell D., Painter T.G. (2004). Aging of oil-impregnated paper in power transformers. IEEE Trans. Power Deliv..

[5] Wang Z. Artificial Intelligence Applications in the Diagnosis of Power Transformer Incipient Faults. https://vtechworks.lib.vt.edu/handle/10919/28594.

[6] Sang Z.X., Mao C.X., Lu J.M., Wang D. (2013). Analysis and simulation of fault characteristics of power switch failures in distribution electronic power transformers. Energies.

[7] Mineral Oil-Impregnated Electrical Equipment in Service—Guide to the Interpretation of Dissolved and Free Gases Analysis. https://www.vde-verlag.de/iec-normen/preview-pdf/info_iec60599%7Bed2.1%7Db.pdf.

[8] Souahlia S., Bacha K., Chaari A. (2012). MLP neural network-based decision for power transformers fault diagnosis using an improved combination of rogers and doernenburg ratios DGA. Int. J. Electr. Power Energy Syst..

[9] Coward H.F., Jones G.W. (1928). Limits of inflammability of gases and vapors. J. Franklin Inst..

[10] Cashdollar K.L., Zlochower I.A., Green G.M., Thomas R.A., Hertzberg M. (2000). Flammability of methane, propane, and hydrogen gases. J. Loss Prev. Process Ind..

[11] Wang L.W., Kang Y.F., Liu X.H., Zhang S.M., Huang W.P., Wang S.R. (2012). ZnO nanorod gas sensor for ethanol detection. Sens. Actuators B Chem..

[12] Meng D., Yamazaki T., Shen Y., Liu Z., Kikuta T. (2009). Preparation of WO_3_ nanoparticles and application to NO_2_ sensor. Appl. Surf. Sci..

[13] Zou X., Li G., Wang P., Su J., Zhao J., Zhou L., Wang Y.N., Chen J. (2012). A precursor route to single-crystalline WO_3_ nano-plates with an uneven surface and enhanced sensing properties. Dalton Trans..

[14] Zhang H., Liu Z., Yang J., Guo W., Zhu L., Zheng W. (2014). Temperature and acidity effects on WO_3_ nanostructures and gas-sensing properties of WO_3_ nanoplates. Mater. Res. Bull..

[15] Wei S., Xing Y., Li Y., Zhao Y., Du W., Zhou M. (2016). Preparation and gas sensing properties of flower-like WO3 hierarchical architecture. Vacuum.

[16] Nikfarjam A., Fardindoost S., Zad A.I. (2013). Fabrication of Pd Doped WO_3_ Nanofiber as Hydrogen Sensor. Polymers.

[17] Hu T., Wang D., Wang M., Li Z., Yang M. (2014). Miniature Hydrogen Sensor Based on Fiber Inner Cavity and Pt-doped WO_3_ Coating. IEEE Photonics Technol. Lett..

[18] Kaur J., Anand K., Kohli N., Kaur A., Singh R.C. (2018). Temperature dependent selective detection of hydrogen and acetone using Pd doped WO_3_/Reduced graphene oxide nanocomposite. Chem. Phys. Lett..

[19] Kuar J., Anand K., Kaur A., Singh R.C. (2017). Sensitive and selective acetone sensor based on Gd doped WO_3_/reduced graphene oxide nanocomposite. Sens. Actuators B Chem..

[20] Shi J., Chenga Z., Gao L., Zhang Y., Xu J., Zhao H. (2016). Facile synthesis of reduced graphene oxide/hexagonal WO_3_ nanosheets composites with enhanced H_2_S sensing properties. Sens. Actuators B Chem..

[21] Chen W., Zhou Q., Gao T., Su X., Wan Fu. (2013). Pd-doped SnO_2_-based sensor detecting characteristic fault hydrocarbon gases in transformer oil. J. Nanomater..

[22] Zhang H. (2018). Hierarchically porous WO_3_ microstructures with networks for acetylene sensing application. Mater. Lett..

[23] Hummers W.S., Offeman R.E. (1958). Preparation of graphitic oxide. J. Am. Chem. Soc..

[24] Liu Y., Koep E., Liu M. (2005). A highly sensitive and fast-responding SnO_2_ sensor fabricated by combustion chemical vapor deposition. Chem. Mater..

[25] Rossi C., Conto T.D., Estève D., Larangot B. (2001). Design, fabrication and modelling of MEMS-based microthrusters for space application. Smart Mater. Struct..

[26] Matsui M. (2001). Thermal Stability of Au Thin Film Deposited on Al_2_O_3_ Substrate with RuO_2_ Adhesion Layer. J. Ceram. Soc. Jpn..

[27] Norris B.J., Anderson J., Wager J.F., Keszler D.A. (2003). Spin-coated zinc oxide transparent transistors. J. Phys. D Appl. Phys..

[28] Zhang M., Lei D., Du Z., Yin X., Chen L., Li Q., Wang Y., Wang T. (2011). Fast synthesis of SnO_2_/graphene composites by reducing graphene oxide with stannous ions. J. Mater. Chem..

[29] Chen X., Kalenczuk R.J., Wajda A., Łapczuk J., Kurzewski M., Drozdzik M., Chu P.K., Palen E.B. (2012). Synthesis, disper-sion, and cytocompatibility of graphene oxide and reduced graphene oxide. Colliods Surf. B.

[30] Upadhyay S.B., Mishra R.K., Sahay P.P. (2015). Enhanced acetone response in co-precipitated WO3 nanostructures upon indium doping. Sens. Actuators B Chem..

[31] Tang L., Wan Y., Yan D., Pei Y., Zhao L., Li Y., Wu L., Jiang J., Lai G. (2013). The effect of graphene dispersion on the mechanical properties of graphene/epoxy composites. Carbon.

[32] Ji Z., Shen X., Zhu G., Zhou H., Yuan A. (2012). Reduced graphene oxide/nickel nanocomposites: Facile synthesis, magnetic and catalytic properties. J. Mater. Chem..

[33] Garciasanchez R.F., Ahmido T., Casimir D., Baliga S., Misra P. (2013). Thermal Effects Associated with the Raman Spectroscopy of WO_3_ Gas-Sensor Materials. J. Phys. Chem. A.

[34] Park O., Hahm M.G., Lee S., Joh H., Na S., Vajtai R., Lee J.H., Ku B.C., Ajayan P.M. (2012). In Situ Synthesis of Thermochemically Reduced Graphene Oxide Conducting Nanocomposites. Nano. Lett..

[35] Merlen A., Buijnsters J.G., Pardanaud C. (2017). A Guide to and Review of the Use of Multiwavelength Raman Spectroscopy for Characterizing Defective Aromatic Carbon Solids: From Graphene to Amorphous Carbons. Coatings.

[36] Feng Q., Li X., Wang J. (2017). Percolation effect of reduced graphene oxide (rGO) on ammonia sensing of rGO-SnO_2_ composite based sensor. Sens. Actuator B.

[37] Dutta D., Wood B.C., Bhide S.Y., Ayappa K.G., Narasimhan S. (2014). Enhanced Gas Adsorption on Graphitic Substrates via Defects and Local Curvature: A Density Functional Theory Study. J. Phys. Chem. C.

[38] Díazreyes J., Dorantes-García V., Pérez-Benítez A., Balderas-López J.A. (2008). Obtaining of films of tungsten trioxide (WO_3_) by resistive heating of a tungsten filament. Superficies Y Vacío.

[39] Hardcastle F.D., Wachs I.E. (1995). Determination of the molecular structures of tungstates by Raman spectroscopy. J. Raman Spectrosc..

[40] Xu T., Zhang L., Cheng H., Zhu Y. (2011). Significantly enhanced photocatalytic performance of ZnO via graphene hybridization and the mechanism study. Appl. Catal. B.

[41] Lo S.S., Huang D. (2010). Morphological variation and raman spectroscopy of zno hollow microspheres prepared by a chemical colloidal process. J. Surf. Colloids.

[42] Zaki M.I., Abdel-Khalik M., Habashy G.M. (1986). Acid-leaching and consequent pore structure and bleaching capacity modifications of egyptian clays. Colloids Surf..

[43] Shojaee M., Nasresfahani S., Sheikhi M.H. (2018). Hydrothermally synthesized Pd-loaded SnO_2_/partiallyreduced graphene oxide nanocomposite for effective detection of carbon monoxide at room temperature. Sens. Actuators B Chem..

[44] Anand K., Singh M.P., Singh O., Kohli N., Singh R.C. (2013). Optical and thermal properties of precursor-controlled graphene–zinc nanocomposites. Mater. Sci. Semicon. Proc..

[45] Lv Y., Zhan W., He Y., Wang Y., Kong X., Kuang O., Xie Z., Zheng L. (2014). MOF-templated synthesis of porous Co_3_O_4_ concave nanocubes with high specific surface area and their gas sensing properties. ACS Appl. Mater. Interfaces.

[46] Penner R.M. (2000). Hybrid electrochemical/chemical synthesis of quantum dots. Acc. Chem. Res..

[47] Feng Z., Guo M., Zhang M. (2013). Hydrothermal preparation and optical properties of orientation-controlled WO_3_ nanorod arrays on ITO substrates. Cryst. Eng. Commun..

[48] Lupan O., Emelchenko G.A., Chai G., Redkin A.N., Gruzintsev A.N., Tiginyanu I.M., Chow L., Ono L.K., Cuenya B.R., Heinrich H. (2010). Synthesis and characterization of ZnO nanowires for nanosensor applications. Mater. Res. Bull..

[49] Zheng H., Ou J., Strano M.S., Kaner R.B., Mitchell A., Kalantar-zadeh K. (2011). Nanostructured tungsten oxide–properties, synthesis, and applications. Adv. Funct. Mater..

[50] Malard L.M., Pimenta M.A., Dresselhaus G., Dresselhaus M.S. (2009). Raman spectroscopy in graphene. Phys. Rep..

[51] Zhou D., Zhu Z., Liu B. (2016). Solvothermal synthesis and characterization of a novel reduced graphene oxide (RGO)/BiVO_4_/SiO_2_, nanocomposites. Mater. Lett..

[52] Yu X., Zhao Z., Sun D., Ren N., Yu J., Yang R., Liu H. (2018). Microwave-assisted hydrothermal synthesis of Sn_3_O_4_, nanosheet/rgo planar heterostructure for efficient photocatalytic hydrogen generation. Appl. Catal. B.

[53] Gao C., Zhou J., Liu G., Wang L. (2017). Microwave-assisted synthesis and surface decoration of lifepo 4, hexagonal nanoplates for lithium-ion batteries with excellent electrochemical performance. J. Mater. Sci..

[54] Zang L.Y., Misra H.P. (1992). Epr kinetic studies of superoxide radicals generated during the autoxidation of 1-methyl-4-phenyl-2,3-dihydropyridinium, a bioactivated intermediate of parkinsonian-inducing neurotoxin 1-methyl-4-phenyl-1,2,3,6-tetrahydropyridine. J. Biol. Chem..

[55] Chen Z., Sun P., Fan B., Zhang Z., Fang X. (2014). In situ template-free ion-exchange process to prepare visible-light-active g-C_3_N_4_/Nis hybrid photocatalysts with enhanced hydrogen evolution activity. J. Phys. Chem. C.

[56] Jin L., Chen W., Zhang H., Xiao G., Yu C., Zhou Q. (2016). Characterization of Reduced Graphene Oxide (rGO)-Loaded SnO_2_ Nanocomposite and Applications in C_2_H_2_ Gas Detection. Appl. Sci..

[57] Uddin A.S.M.I., Lee K.W., Chung G.S. (2015). Acetylene gas sensing properties of an Ag-loaded hierarchical ZnO nanostructure-decorated reduced graphene oxide hybrid. Sens. Actuators B Chem..

[58] Uddin A.S.M.I., Chung G.S. (2014). Synthesis of highly dispersed ZnO nanoparticles on graphene surface and their acetylene sensing properties. Sens. Actuators B Chem..

[59] Liewhiran C., Tamaekong N., Wisitsoraat A., Phanichphant S. (2012). Highly selective environmental sensors based on flame-spray-made SnO_2_ nanoparticles. Sens. Actuators B Chem..

[60] Tamaekong N., Liewhiran C., Wisitsoraat A., Phanichphant S. (2011). Acetylene sensor based on Pt/ZnO thick films as prepared by flame spray pyrolysis. Sens. Actuators B Chem..

[61] Lee K.W., Uddin A.S.M.I., Phan D.T., Chung G.S. (2015). Fabrication of low-temperature acetylene gas sensor based on Ag nanoparticles-loaded hierarchical ZnO nanostructures. Electron. Lett..

[62] Tammanoon N., Wisitsoraat A., Sriprachuabwong C., Phokharatkul D., Tuantranont A., Phanichphant S., Liewhiran C. (2015). Ultrasensitive NO_2_ sensor based on ohmic metal–semiconductor interfaces of electrolytically exfoliated graphene/flame-spray-made SnO_2_ nanoparticles composite operating at low temperatures. ACS Appl. Mater. Interfaces.

[63] Garg R., Dutta N.K., Choudhury N.R. (2014). Work function engineering of graphene. Nanomaterials.

[64] Quang P.L., Cuong N.D., Hoa T.T., Long H.T., Chu M.H., Le D.T.T., Hieu N.Y. (2018). Simple post-synthesis of mesoporous p-type Co_3_O_4_, nanochains for enhanced H2S gas sensing performance. Sens. Actuators B Chem..

[65] Acharya S., Bangera K.V., Shivakumar G.K. (2016). Conduction mechanism in n-CdSe/p-ZnTe heterojunction. J. Electron. Mater..

[66] Chen W.G., Gao T.Y., Li Q.Z., Gan H.I. (2014). Enhanced gas sensing properties of flower-like ZnO nanostructure to acetylene. Mater. Process. Rep..

